# Methods for the synthesis of qualitative research: a critical review

**DOI:** 10.1186/1471-2288-9-59

**Published:** 2009-08-11

**Authors:** Elaine Barnett-Page, James Thomas

**Affiliations:** 1 Evidence for Policy and Practice Information and Co-ordinating (EPPI-) Centre, Social Science Research Unit, 18 Woburn Square, London WC1H 0NS, UK

## Abstract

**Background:**

In recent years, a growing number of methods for synthesising qualitative research have emerged, particularly in relation to health-related research. There is a need for both researchers and commissioners to be able to distinguish between these methods and to select which method is the most appropriate to their situation.

**Discussion:**

A number of methodological and conceptual links between these methods were identified and explored, while contrasting epistemological positions explained differences in approaches to issues such as quality assessment and extent of iteration. Methods broadly fall into 'realist' or 'idealist' epistemologies, which partly accounts for these differences.

**Summary:**

Methods for qualitative synthesis vary across a range of dimensions. Commissioners of qualitative syntheses might wish to consider the kind of product they want and select their method – or type of method – accordingly.

## Background

The range of different methods for synthesising qualitative research has been growing over recent years [1,2], alongside an increasing interest in qualitative synthesis to inform health-related policy and practice [3]. While the terms 'meta-analysis' (a statistical method to combine the results of primary studies), or sometimes 'narrative synthesis', are frequently used to describe how quantitative research is synthesised, far more terms are used to describe the synthesis of qualitative research. This profusion of terms can mask some of the basic similarities in approach that the different methods share, and also lead to some confusion regarding which method is most appropriate in a given situation. This paper does not argue that the various nomenclatures are unnecessary, but rather seeks to draw together and review the full range of methods of synthesis available to assist future reviewers in selecting a method that is fit for their purpose. It also represents an attempt to guide the reader through some of the varied terminology to spring up around qualitative synthesis. Other helpful reviews of synthesis methods have been undertaken in recent years with slightly different foci to this paper. Two recent studies have focused on describing and critiquing methods for the integration of qualitative research with quantitative [4,5] rather than exclusively examining the detail and rationale of methods for the synthesis of qualitative research. Two other significant pieces of work give practical advice for conducting the synthesis of qualitative research, but do not discuss the full range of methods available [6,7]. We begin our Discussion by outlining each method of synthesis in turn, before comparing and contrasting characteristics of these different methods across a range of dimensions. Readers who are more familiar with the synthesis methods described here may prefer to turn straight to the 'dimensions of difference' analysis in the second part of the Discussion.

## Discussion

### Overview of synthesis methods

#### Meta-ethnography

In their seminal work of 1988, Noblit and Hare proposed meta-ethnography as an alternative to meta-analysis [8]. They cited Strike and Posner's [9] definition of synthesis as an activity in which separate parts are brought together to form a 'whole'; this construction of the whole is essentially characterised by some degree of innovation, so that the result is greater than the sum of its parts. They also borrowed from Turner's theory of social explanation [10], a key tenet of which was building 'comparative understanding' [[8], p22] rather than aggregating data.

To Noblit and Hare, synthesis provided an answer to the question of 'how to "put together" written interpretive accounts' [[8], p7], where mere integration would not be appropriate. Noblit and Hare's early work synthesised research from the field of education.

Three different methods of synthesis are used in meta-ethnography. One involves the 'translation' of concepts from individual studies into one another, thereby evolving overarching concepts or metaphors. Noblit and Hare called this process *reciprocal translational analysis *(RTA). *Refutational *synthesis involves exploring and explaining contradictions between individual studies. *Lines-of-argument *(LOA) synthesis involves building up a picture of the whole (i.e. culture, organisation etc) from studies of its parts. The authors conceptualised this latter approach as a type of grounded theorising.

Britten et al [11] and Campbell et al [12] have both conducted evaluations of meta-ethnography and claim to have succeeded, by using this method, in producing theories with greater explanatory power than could be achieved in a narrative literature review. While both these evaluations used small numbers of studies, more recently Pound et al [13] conducted both an RTA and an LOA synthesis using a much larger number of studies (37) on resisting medicines. These studies demonstrate that meta-ethnography has evolved since Noblit and Hare first introduced it. Campbell et al claim to have applied the method successfully to non-ethnographical studies. Based on their reading of Schutz [14], Britten et al have developed both second and third order constructs in their synthesis (Noblit and Hare briefly allude to the possibility of a 'second level of synthesis' [[8], p28] but do not demonstrate or further develop the idea).

In a more recent development, Sandelowski & Barroso [15] write of adapting RTA by using it to '*integrate *findings interpretively, as opposed to comparing them interpretively' (p204). The former would involve looking to see whether the same concept, theory etc exists in different studies; the latter would involve the construction of a bigger picture or theory (i.e. LOA synthesis). They also talk about comparing or integrating imported concepts (e.g. from other disciplines) as well as those evolved 'in vivo'.

#### Grounded theory

Kearney [16], Eaves [17] and Finfgeld [18] have all adapted grounded theory to formulate a method of synthesis. Key methods and assumptions of grounded theory, as originally formulated and subsequently refined by Glaser and Strauss [19] and Strauss and Corbin [20,21], include: simultaneous phases of data collection and analysis; an inductive approach to analysis, allowing the theory to emerge from the data; the use of the constant comparison method; the use of theoretical sampling to reach theoretical saturation; and the generation of new theory. Eaves cited grounded theorists Charmaz [22] and Chesler [23], as well as Strauss and Corbin [20], as informing her approach to synthesis.

Glaser and Strauss [19] foresaw a time when a substantive body of grounded research should be pushed towards a higher, more abstract level. As a piece of methodological work, Eaves undertook her own synthesis of the synthesis methods used by these authors to produce her own clear and explicit guide to synthesis in grounded formal theory. Kearney stated that 'grounded formal theory', as she termed this method of synthesis, 'is suited to study of phenomena involving processes of contextualized understanding and action' [[24], p180] and, as such, is particularly applicable to nurses' research interests.

As Kearney suggested, the examples examined here were largely dominated by research in nursing. Eaves synthesised studies on care-giving in rural African-American families for elderly stroke survivors; Finfgeld on courage among individuals with long-term health problems; Kearney on women's experiences of domestic violence.

Kearney explicitly chose 'grounded formal theory' because it matches 'like' with 'like': that is, it applies the same methods that have been used to generate the original grounded theories included in the synthesis – produced by constant comparison and theoretical sampling – to generate a higher-level grounded theory. The wish to match 'like' with 'like' is also implicit in Eaves' paper. This distinguishes grounded formal theory from more recent applications of meta-ethnography, which have sought to include qualitative research using diverse methodological approaches [12].

#### Thematic Synthesis

Thomas and Harden [25] have developed an approach to synthesis which they term 'thematic synthesis'. This combines and adapts approaches from both meta-ethnography and grounded theory. The method was developed out of a need to conduct reviews that addressed questions relating to intervention need, appropriateness and acceptability – as well as those relating to effectiveness – without compromising on key principles developed in systematic reviews. They applied thematic synthesis in a review of the barriers to, and facilitators of, healthy eating amongst children.

Free codes of findings are organised into 'descriptive' themes, which are then further interpreted to yield 'analytical' themes. This approach shares characteristics with later adaptations of meta-ethnography, in that the analytical themes are comparable to 'third order interpretations' and that the development of descriptive and analytical themes using coding invoke reciprocal 'translation'. It also shares much with grounded theory, in that the approach is inductive and themes are developed using a 'constant comparison' method. A novel aspect of their approach is the use of computer software to code the results of included studies line-by-line, thus borrowing another technique from methods usually used to analyse primary research.

#### Textual Narrative Synthesis

Textual narrative synthesis is an approach which arranges studies into more homogenous groups. Lucas et al [26] comment that it has proved useful in synthesising evidence of different types (qualitative, quantitative, economic etc). Typically, study characteristics, context, quality and findings are reported on according to a standard format and similarities and differences are compared across studies. Structured summaries may also be developed, elaborating on and putting into context the extracted data [27].

Lucas et al [26] compared thematic synthesis with textual narrative synthesis. They found that 'thematic synthesis holds most potential for hypothesis generation' whereas textual narrative synthesis is more likely to make transparent heterogeneity between studies (as does meta-ethnography, with refutational synthesis) and issues of quality appraisal. This is possibly because textual narrative synthesis makes clearer the context and characteristics of each study, while the thematic approach organises data according to themes. However, Lucas et al found that textual narrative synthesis is 'less good at identifying commonality' (p2); the authors do not make explicit why this should be, although it may be that organising according to themes, as the thematic approach does, is comparatively more successful in revealing commonality.

#### Meta-study

Paterson et al [28] have evolved a multi-faceted approach to synthesis, which they call 'meta-study'. The sociologist Zhao [29], drawing on Ritzer's work [30], outlined three components of analysis, which they proposed should be undertaken prior to synthesis. These are meta-data-analysis (the analysis of findings), meta-method (the analysis of methods) and meta-theory (the analysis of theory). Collectively, these three elements of analysis, culminating in synthesis, make up the practice of 'meta-study'. Paterson et al pointed out that the different components of analysis may be conducted concurrently.

Paterson et al argued that primary research is a construction; secondary research is therefore a construction of a construction. There is need for an approach that recognises this, and that also recognises research to be a product of its social, historical and ideological context. Such an approach would be useful in accounting for differences in research findings. For Paterson et al, there is no such thing as 'absolute truth'.

Meta-study was developed to study the experiences of adults living with a chronic illness. Meta-data-analysis was conceived of by Paterson et al in similar terms to Noblit and Hare's meta-ethnography (see above), in that it is essentially interpretive and seeks to reveal similarities and discrepancies among accounts of a particular phenomenon. Meta-method involves the examination of the methodologies of the individual studies under review. Part of the process of meta-method is to consider different aspects of methodology such as sampling, data collection, research design etc, similar to procedures others have called 'critical appraisal' (CASP [31]). However, Paterson et al take their critique to a deeper level by establishing the underlying assumptions of the methodologies used and the relationship between research outcomes and methods used. Meta-theory involves scrutiny of the philosophical and theoretical assumptions of the included research papers; this includes looking at the wider context in which new theory is generated. Paterson et al described meta-synthesis as a process which creates a new interpretation which accounts for the results of all three elements of analysis. The process of synthesis is iterative and reflexive and the authors were unwilling to oversimplify the process by 'codifying' procedures for bringing all three components of analysis together.

#### Meta-narrative

Greenhalgh et al [32]'s meta-narrative approach to synthesis arose out of the need to synthesise evidence to inform complex policy-making questions and was assisted by the formation of a multi-disciplinary team. Their approach to review was informed by Thomas Kuhn's *The Structure of Scientific Revolutions *[33], in which he proposed that knowledge is produced within particular paradigms which have their own assumptions about theory, about what is a legitimate object of study, about what are legitimate research questions and about what constitutes a finding. Paradigms also tend to develop through time according to a particular set of stages, central to which is the stage of 'normal science', in which the particular standards of the paradigm are largely unchallenged and seen to be self-evident. As Greenhalgh et al pointed out, Kuhn saw paradigms as largely incommensurable: 'that is, an empirical discovery made using one set of concepts, theories, methods and instruments cannot be satisfactorily explained through a different paradigmatic lens' [[32], p419].

Greenhalgh et al synthesised research from a wide range of disciplines; their research question related to the diffusion of innovations in health service delivery and organisation. They thus identified a need to synthesise findings from research which contains many different theories arising from many different disciplines and study designs.

Based on Kuhn's work, Greenhalgh et al proposed that, across different paradigms, there were multiple – and potentially mutually contradictory – ways of understanding the concept at the heart of their review, namely the diffusion of innovation. Bearing this in mind, the reviewers deliberately chose to select key papers from a number of different research 'paradigms' or 'traditions', both within and beyond healthcare, guided by their multidisciplinary research team. They took as their unit of analysis the 'unfolding "storyline" of a research tradition over time' [[32], p417) and sought to understand diffusion of innovation as it was conceptualised in each of these traditions. Key features of each tradition were mapped: historical roots, scope, theoretical basis; research questions asked and methods/instruments used; main empirical findings; historical development of the body of knowledge (how have earlier findings led to later findings); and strengths and limitations of the tradition. The results of this exercise led to maps of 13 'meta-narratives' in total, from which seven key dimensions, or themes, were identified and distilled for the synthesis phase of the review.

#### Critical Interpretive Synthesis

Dixon-Woods et al [34] developed their own approach to synthesising multi-disciplinary and multi-method evidence, termed 'critical interpretive synthesis', while researching access to healthcare by vulnerable groups. Critical interpretive synthesis is an adaptation of meta-ethnography, as well as borrowing techniques from grounded theory. The authors stated that they needed to adapt traditional meta-ethnographic methods for synthesis, since these had never been applied to quantitative as well as qualitative data, nor had they been applied to a substantial body of data (in this case, 119 papers).

Dixon-Woods et al presented critical interpretive synthesis as an approach to the whole process of review, rather than to just the synthesis component. It involves an iterative approach to refining the research question and searching and selecting from the literature (using theoretical sampling) and defining and applying codes and categories. It also has a particular approach to appraising quality, using relevance – i.e. likely contribution to theory development – rather than methodological characteristics as a means of determining the 'quality' of individual papers [35]. The authors also stress, as a defining characteristic, critical interpretive synthesis's critical approach to the literature in terms of deconstructing research traditions or theoretical assumptions as a means of contextualising findings.

Dixon-Woods et al rejected reciprocal translational analysis (RTA) as this produced 'only a summary in terms that have already been used in the literature' [[34], p5], which was seen as less helpful when dealing with a large and diverse body of literature. Instead, Dixon-Woods et al adopted a lines-of-argument (LOA) synthesis, in which – rejecting the difference between first, second and third order constructs – they instead developed 'synthetic constructs' which were then linked with constructs arising directly from the literature.

The influence of grounded theory can be seen in particular in critical interpretive synthesis's inductive approach to formulating the review question and to developing categories and concepts, rejecting a 'stage' approach to systematic reviewing, and in selecting papers using theoretical sampling. Dixon-Woods et al also claim that critical interpretive synthesis is distinct in its 'explicit orientation towards theory generation' [[34], p9].

#### Ecological Triangulation

Jim Banning is the author of 'ecological triangulation' or 'ecological sentence synthesis', applying this method to the evidence for what works for youth with disabilities. He borrows from Webb et al [36] and Denzin [37] the concept of triangulation, in which phenomena are studied from a variety of vantage points. His rationale is that building an 'evidence base' of effectiveness requires the synthesis of cumulative, multi-faceted evidence in order to find out 'what intervention works for what kind of outcomes for what kind of persons under what kind of conditions' [[38], p1].

Ecological triangulation unpicks the mutually interdependent relationships between behaviour, persons and environments. The method requires that, for data extraction and synthesis, 'ecological sentences' are formulated following the pattern: 'With this intervention, these outcomes occur with these population foci and within these grades (ages), with these genders ... and these ethnicities in these settings' [[39], p1].

#### Framework Synthesis

Brunton et al [40] and Oliver et al [41] have applied a 'framework synthesis' approach in their reviews. Framework synthesis is based on framework analysis, which was outlined by Pope, Ziebland and Mays [42], and draws upon the work of Ritchie and Spencer [43] and Miles and Huberman [44]. Its rationale is that qualitative research produces large amounts of textual data in the form of transcripts, observational fieldnotes etc. The sheer wealth of information poses a challenge for rigorous analysis. Framework synthesis offers a highly structured approach to organising and analysing data (e.g. indexing using numerical codes, rearranging data into charts etc).

Brunton et al applied the approach to a review of children's, young people's and parents' views of walking and cycling; Oliver et al to an analysis of public involvement in health services research. Framework synthesis is distinct from the other methods outlined here in that it utilises an *a priori *'framework' – informed by background material and team discussions – to extract and synthesise findings. As such, it is largely a deductive approach although, in addition to topics identified by the framework, new topics may be developed and incorporated as they emerge from the data. The synthetic product can be expressed in the form of a chart for each key dimension identified, which may be used to map the nature and range of the concept under study and find associations between themes and exceptions to these [40].

#### 'Fledgling' approaches

There are three other approaches to synthesis which have not yet been widely used. One is an approach using content analysis [45,46] in which text is condensed into fewer content-related categories. Another is 'meta-interpretation' [47], featuring the following: an ideographic rather than pre-determined approach to the development of exclusion criteria; a focus on meaning in context; interpretations as raw data for synthesis (although this feature doesn't distinguish it from other synthesis methods); an iterative approach to the theoretical sampling of studies for synthesis; and a transparent audit trail demonstrating the trustworthiness of the synthesis.

In addition to the synthesis methods discussed above, Sandelowski and Barroso propose a method they call 'qualitative metasummary' [15]. It is mentioned here as a new and original approach to handling a collection of qualitative studies but is qualitatively different to the other methods described here since it is aggregative; that is, findings are accumulated and summarised rather than 'transformed'. Metasummary is a way of producing a 'map' of the contents of qualitative studies and – according to Sandelowski and Barroso – 'reflect [s] a quantitative logic' [[15], p151]. The frequency of each finding is determined and the higher the frequency of a particular finding, the greater its validity. The authors even discuss the calculation of 'effect sizes' for qualitative findings. Qualitative metasummaries can be undertaken as an end in themselves or may serve as a basis for a further synthesis.

### Dimensions of difference

Having outlined the range of methods identified, we now turn to an examination of how they compare with one another. It is clear that they have come from many different contexts and have different approaches to understanding knowledge, but what do these differences mean in practice? Our framework for this analysis is shown in Additional file 1: *dimensions of difference *[48]. We have examined the epistemology of each of the methods and found that, to some extent, this explains the need for different methods and their various approaches to synthesis.

#### Epistemology

The first dimension that we will consider is that of the researchers' epistemological assumptions. Spencer et al [49] outline a range of epistemological positions, which might be organised into a spectrum as follows:

*Subjective idealism*: there is no shared reality independent of multiple alternative human constructions

*Objective idealism*: there is a world of collectively shared understandings

*Critical realism*: knowledge of reality is mediated by our perceptions and beliefs

*Scientific realism*: it is possible for knowledge to approximate closely an external reality

*Naïve realism*: reality exists independently of human constructions and can be known directly [49,45,46].

Thus, at one end of the spectrum we have a highly constructivist view of knowledge and, at the other, an unproblematized 'direct window onto the world' view.

Nearly all of positions along this spectrum are represented in the range of methodological approaches to synthesis covered in this paper. The originators of meta-narrative synthesis, critical interpretive synthesis and meta-study all articulate what might be termed a 'subjective idealist' approach to knowledge. Paterson et al [28] state that meta-study shies away from creating 'grand theories' within the health or social sciences and assume that no single objective reality will be found. Primary studies, they argue, are themselves constructions; meta-synthesis, then, 'deals with constructions of constructions' (p7). Greenhalgh et al [32] also view knowledge as a product of its disciplinary paradigm and use this to explain conflicting findings: again, the authors neither seek, nor expect to find, one final, non-contestable answer to their research question. Critical interpretive synthesis is similar in seeking to place literature within its context, to question its assumptions and to produce a theoretical model of a phenomenon which – because highly interpretive – may not be reproducible by different research teams at alternative points in time [[34], p11].

Methods used to synthesise grounded theory studies in order to produce a higher level of grounded theory [24] appear to be informed by 'objective idealism', as does meta-ethnography. Kearney argues for the near-universal applicability of a 'ready-to-wear' theory across contexts and populations. This approach is clearly distinct from one which recognises multiple realities. The emphasis is on examining commonalities amongst, rather than discrepancies between, accounts. This emphasis is similarly apparent in most meta-ethnographies, which are conducted either according to Noblit and Hare's 'reciprocal translational analysis' technique or to their 'lines-of-argument' technique and which seek to provide a 'whole' which has a greater explanatory power. Although Noblit and Hare also propose 'refutational synthesis', in which contradictory findings might be explored, there are few examples of this having been undertaken in practice, and the aim of the method appears to be to explain and explore differences due to context, rather than multiple realities.

Despite an assumption of a reality which is perhaps less contestable than those of meta-narrative synthesis, critical interpretive synthesis and meta-study, both grounded formal theory and meta-ethnography place a great deal of emphasis on the interpretive nature of their methods. This still supposes a degree of constructivism. Although less explicit about how their methods are informed, it seems that both thematic synthesis and framework synthesis – while also involving some interpretation of data – share an even less problematized view of reality and a greater assumption that their synthetic products are reproducible and correspond to a shared reality. This is also implicit in the fact that such products are designed directly to inform policy and practice, a characteristic shared by ecological triangulation. Notably, ecological triangulation, according to Banning, can be either realist or idealist. Banning argues that the interpretation of triangulation can either be one in which multiple viewpoints converge on a point to produce confirming evidence (i.e. one definitive answer to the research question) or an idealist one, in which the complexity of multiple viewpoints is represented. Thus, although ecological triangulation views reality as complex, the approach assumes that it can be approximately knowable (at least when the realist view of ecological triangulation is adopted) and that interventions can and should be modelled according to the products of its syntheses.

While pigeonholing different methods into specific epistemological positions is a problematic process, we do suggest that the contrasting epistemologies of different researchers is one way of explaining why we have – and need – different methods for synthesis.

#### Iteration

Variation in terms of the extent of iteration during the review process is another key dimension. All synthesis methods include some iteration but the degree varies. Meta-ethnography, grounded theory and thematic synthesis all include iteration at the synthesis stage; both framework synthesis and critical interpretive synthesis involve iterative literature searching – in the case of critical interpretive synthesis, it is not clear whether iteration occurs during the rest of the review process. Meta-narrative also involves iteration at every stage. Banning does not mention iteration in outlining ecological triangulation and neither do Lucas or Thomas and Harden for thematic narrative synthesis.

It seems that the more idealist the approach, the greater the extent of iteration. This might be because a large degree of iteration does not sit well with a more 'positivist' ideal of procedural objectivity; in particular, the notion that the robustness of the synthetic product depends in part on the reviewers stating up front in a protocol their searching strategies, inclusion/exclusion criteria etc, and being seen not to alter these at a later stage.

#### Quality assessment

Another dimension along which we can look at different synthesis methods is that of quality assessment. When the approaches to the assessment of the quality of studies retrieved for review are examined, there is again a wide methodological variation. It might be expected that the further towards the 'realism' end of the epistemological spectrum a method of synthesis falls, the greater the emphasis on quality assessment. In fact, this is only partially the case.

Framework synthesis, thematic narrative synthesis and thematic synthesis – methods which might be classified as sharing a 'critical realist' approach – all have highly specified approaches to quality assessment. The review in which framework synthesis was developed applied ten quality criteria: two on quality and reporting of sampling methods, four to the quality of the description of the sample in the study, two to the reliability and validity of the tools used to collect data and one on whether studies used appropriate methods for helping people to express their views. Studies which did not meet a certain number of quality criteria were excluded from contributing to findings. Similarly, in the example review for thematic synthesis, 12 criteria were applied: five related to reporting aims, context, rationale, methods and findings; four relating to reliability and validity; and three relating to the appropriateness of methods for ensuring that findings were rooted in participants' own perspectives. Studies which were deemed to have significant flaws were excluded and sensitivity analyses were used to assess the possible impact of study quality on the review's findings. Thomas and Harden's use of thematic narrative synthesis similarly applied quality criteria and developed criteria additional to those they found in the literature on quality assessment, relating to the extent to which people's views and perspectives had been privileged by researchers. It is worth noting not only that these methods apply quality criteria but that they are explicit about what they are: assessing quality is a key component in the review process for both of these methods. Likewise, Banning – the originator of ecological triangulation – sees quality assessment as important and adapts the Design and Implementation Assessment Device (DIAD) Version 0.3 (a quality assessment tool for quantitative research) for use when appraising qualitative studies [50]. Again, Banning writes of excluding studies deemed to be of poor quality.

Greenhalgh et al's meta-narrative review [32] modified a range of existing quality assessment tools to evaluate studies according to validity and robustness of methods; sample size and power; and validity of conclusions. The authors imply, but are not explicit, that this process formed the basis for the exclusion of some studies. Although not quite so clear about quality assessment methods as framework and thematic synthesis, it might be argued that meta-narrative synthesis shows a greater commitment to the concept that research can and should be assessed for quality than either meta-ethnography or grounded formal theory. The originators of meta-ethnography, Noblit and Hare [8], originally discussed quality in terms of quality of metaphor, while more recent use of this method has used amended versions of CASP (the Critical Appraisal Skills Programme tool, [31]), yet has only referred to studies being excluded on the basis of lack of relevance or because they weren't 'qualitative' studies [8]. In grounded theory, quality assessment is only discussed in terms of a 'personal note' being made on the context, quality and usefulness of each study. However, contrary to expectation, meta-narrative synthesis lies at the extreme end of the idealism/realism spectrum – as a subjective idealist approach – while meta-ethnography and grounded theory are classified as objective idealist approaches.

Finally, meta-study and critical interpretive synthesis – two more subjective idealist approaches – look to the content and utility of findings rather than methodology in order to establish quality. While earlier forms of meta-study included only studies which demonstrated 'epistemological soundness', in its most recent form [51] this method has sought to include all relevant studies, excluding only those deemed not to be 'qualitative' research. Critical interpretive synthesis also conforms to what we might expect of its approach to quality assessment: quality of research is judged as the extent to which it informs theory. The threshold of inclusion is informed by expertise and instinct rather than being articulated a priori.

In terms of quality assessment, it might be important to consider the academic context in which these various methods of synthesis developed. The reason why thematic synthesis, framework synthesis and ecological triangulation have such highly specified approaches to quality assessment may be that each of these was developed for a particular task, i.e. to conduct a multi-method review in which randomised controlled trials (RCTs) were included. The concept of quality assessment in relation to RCTs is much less contested and there is general agreement on criteria against which quality should be judged.

#### Problematizing the literature

Critical interpretive synthesis, the meta-narrative approach and the meta-theory element of meta-study all share some common ground in that their review and synthesis processes include examining all aspects of the context in which knowledge is produced. In conducting a review on access to healthcare by vulnerable groups, critical interpretive synthesis sought to question 'the ways in which the literature had constructed the problematics of access, the nature of the assumptions on which it drew, and what has influenced its choice of proposed solutions' [[34], p6]. Although not claiming to have been directly influenced by Greenhalgh et al's meta-narrative approach, Dixon-Woods et al do cite it as sharing similar characteristics in the sense that it critiques the literature it reviews.

Meta-study uses meta-theory to describe and deconstruct the theories that shape a body of research and to assess its quality. One aspect of this process is to examine the historical evolution of each theory and to put it in its socio-political context, which invites direct comparison with meta-narrative synthesis. Greenhalgh et al put a similar emphasis on placing research findings within their social and historical context, often as a means of seeking to explain heterogeneity of findings. In addition, meta-narrative shares with critical interpretive synthesis an iterative approach to searching and selecting from the literature.

Framework synthesis, thematic synthesis, textual narrative synthesis, meta-ethnography and grounded theory do not share the same approach to problematizing the literature as critical interpretive synthesis, meta-study and meta-narrative. In part, this may be explained by the extent to which studies included in the synthesis represented a broad range of approaches or methodologies. This, in turn, may reflect the broadness of the review question and the extent to which the concepts contained within the question are pre-defined within the literature. In the case of both the critical interpretive synthesis and meta-narrative reviews, terminology was elastic and/or the question formed iteratively. Similarly, both reviews placed great emphasis on employing multi-disciplinary research teams. Approaches which do not critique the literature in the same way tend to have more narrowly-focused questions. They also tend to include a more limited range of studies: grounded theory synthesis includes grounded theory studies, meta-ethnography (in its original form, as applied by Noblit and Hare) ethnographies. The thematic synthesis incorporated studies based on only a narrow range of qualitative methodologies (interviews and focus groups) which were informed by a similarly narrow range of epistemological assumptions. It may be that the authors of such syntheses saw no need for including such a critique in their review process.

#### Similarities and differences between primary studies

Most methods of synthesis are applicable to heterogeneous data (i.e. studies which use contrasting methodologies) apart from early meta-ethnography and synthesis informed by grounded theory. All methods of synthesis state that, at some level, studies are compared; many are not so explicit about how this is done, though some are. Meta-ethnography is one of the most explicit: it describes the act of 'translation' where terms and concepts which have resonance with one another are subsumed into 'higher order constructs'. Grounded theory, as represented by Eaves [17], is undertaken according to a long list of steps and sub-steps, includes the production of generalizations about concepts/categories, which comes from classifying these categories. In meta-narrative synthesis, comparable studies are grouped together at the appraisal phase of review.

Perhaps more interesting are the ways in which differences between studies are explored. Those methods with a greater emphasis on critical appraisal may tend (although this is not always made explicit) to use differences in method to explain differences in finding. Meta-ethnography proposes 'refutational synthesis' to explain differences, although there are few examples of this in the literature. Some synthesis methods – for example, thematic synthesis – look at other characteristics of the studies under review, whether types of participants and their context vary, and whether this can explain differences in perspective.

All of these methods, then, look within the studies to explain differences. Other methods look beyond the study itself to the context in which it was produced. Critical interpretive synthesis and meta-study look at differences in theory or in socio-economic context. Critical interpretive synthesis, like meta-narrative, also explores epistemological orientation. Meta-narrative is unique in concerning itself with disciplinary paradigm (i.e. the story of the discipline as it progresses). It is also distinctive in that it treats conflicting findings as 'higher order data' [[32], p420], so that the main emphasis of the synthesis appears to be on examining and explaining contradictions in the literature.

#### Going 'beyond' the primary studies

Synthesis is sometimes defined as a process resulting in a product, a 'whole', which is more than the sum of its parts. However, the methods reviewed here vary in the extent to which they attempt to 'go beyond' the primary studies and transform the data. Some methods – textual narrative synthesis, ecological triangulation and framework synthesis – focus on describing and summarising their primary data (often in a highly structured and detailed way) and translating the studies into one another. Others – meta-ethnography, grounded theory, thematic synthesis, meta-study, meta-narrative and critical interpretive synthesis – seek to push beyond the original data to a fresh interpretation of the phenomena under review. A key feature of thematic synthesis is its clear differentiation between these two stages.

Different methods have different mechanisms for going beyond the primary studies, although some are more explicit than others about what these entail. Meta-ethnography proposes a 'Line of Argument' (LOA) synthesis in which an interpretation is constructed to both link and explain a set of parts. Critical interpretive synthesis based its synthesis methods on those of meta-ethnography, developing an LOA using what the authors term 'synthetic constructs' (akin to 'third order constructs' in meta-ethnography) to create a 'synthesising argument'. Dixon-Woods et al claim that this is an advance on Britten et al's methods, in that they reject the difference between first, second and third order constructs.

Meta-narrative, as outlined above, focuses on conflicting findings and constructs theories to explain these in terms of differing paradigms. Meta study derives questions from each of its three components to which it subjects the dataset and inductively generates a number of theoretical claims in relation to it. According to Eaves' model of grounded theory [17], mini-theories are integrated to produce an explanatory framework. In ecological triangulation, the 'axial' codes – or second level codes evolved from the initial deductive open codes – are used to produce Banning's 'ecological sentence' [39].

#### The synthetic product

In overviewing and comparing different qualitative synthesis methods, the ultimate question relates to the utility of the synthetic product: what is it for? It is clear that some methods of synthesis – namely, thematic synthesis, textual narrative synthesis, framework synthesis and ecological triangulation – view themselves as producing an output that is directly applicable to policy makers and designers of interventions. The example of framework synthesis examined here (on children's, young people's and parents' views of walking and cycling) involved policy makers and practitioners in directing the focus of the synthesis and used the themes derived from the synthesis to infer what kind of interventions might be most effective in encouraging walking and cycling. Likewise, the products of the thematic synthesis took the form of practical recommendations for interventions (e.g. 'do not promote fruit and vegetables in the same way in the same intervention'). The extent to which policy makers and practitioners are involved in informing either synthesis or recommendation is less clear from the documents published on ecological triangulation, but the aim certainly is to directly inform practice.

The outputs of synthesis methods which have a more constructivist orientation – meta-study, meta-narrative, meta-ethnography, grounded theory, critical interpretive synthesis – tend to look rather different. They are generally more complex and conceptual, sometimes operating on the symbolic or metaphorical level, and requiring a further process of interpretation by policy makers and practitioners in order for them to inform practice. This is not to say, however, that they are not useful for practice, more that they are doing different work. However, it may be that, in the absence of further interpretation, they are more useful for informing other researchers and theoreticians.

#### Looking across dimensions

After examining the dimensions of difference of our included methods, what picture ultimately emerges? It seems clear that, while similar in some respects, there are genuine differences in approach to the synthesis of what is essentially textual data. To some extent, these differences can be explained by the epistemological assumptions that underpin each method. Our methods split into two broad camps: the idealist and the realist (see Table 1 for a summary). Idealist approaches generally tend to have a more iterative approach to searching (and the review process), have less a priori quality assessment procedures and are more inclined to problematize the literature. Realist approaches are characterised by a more linear approach to searching and review, have clearer and more well-developed approaches to quality assessment, and do not problematize the literature.

**Table 1:** Summary table

	Idealist	Realist
Searching	Iterative	Linear
Quality assessment	Less clear, less a priori; quality of content rather than method	Clear and a priori
Problematizing the literature	Yes	No
Question	Explore	Answer
Heterogeneity	Lots	Little
Synthetic product	Complex	Clear for policy makers and practitioners

N.B.: In terms of the above dimensions, it is generally a question of degree rather than of absolute distinctions.

#### Mapping the relationships between methods

What is interesting is the relationship between these methods of synthesis, the conceptual links between them, and the extent to which the originators cite – or, in some cases, don't cite – one another. Some methods directly build on others – framework synthesis builds on framework analysis, for example, while grounded theory and constant comparative analysis build on grounded theory. Others further develop existing methods – meta-study, critical interpretive synthesis and meta-narrative all adapt aspects of meta-ethnography, while also importing concepts from other theorists (critical interpretive synthesis also adapts grounded theory techniques).

Some methods share a clear conceptual link, without directly citing one another: for example, the analytical themes developed during thematic synthesis are comparable to the third order interpretations of meta-ethnography. The meta-theory aspect of meta-study is echoed in both meta-narrative synthesis and critical interpretive synthesis (see 'Problematizing the literature, above); however, the originators of critical interpretive synthesis only refer to the originators of meta-study in relation to their use of sampling techniques.

## Summary

While methods for qualitative synthesis have many similarities, there are clear differences in approach between them, many of which can be explained by taking account of a given method's epistemology.

However, *within *the two broad idealist/realist categories, any differences between methods in terms of outputs appear to be small.

Since many systematic reviews are designed to inform policy and practice, it is important to select a method – or type of method – that will produce the kind of conclusions needed. However, it is acknowledged that this is not always simple or even possible to achieve in practice.

The approaches that result in more easily translatable messages for policy-makers and practitioners may appear to be more attractive than the others; but we do need to take account lessons from the more idealist end of the spectrum, that some perspectives are not universal.

## Competing interests

The authors declare that they have no competing interests.

## Authors' contributions

Both authors made substantial contributions, with EBP taking a lead on writing and JT on the analytical framework. Both authors read and approved the final manuscript.

## Pre-publication history

The pre-publication history for this paper can be accessed here:

http://www.biomedcentral.com/1471-2288/9/59/prepub

## Supplementary Material

Additional file 1 **Dimensions of difference**. Ranging from subjective idealism through objective idealism and critical realism to scientific realism to naïve realism Click here for file
