# Supplementary material for: Methods for the synthesis of qualitative research: a critical review
Source: BMC Med Res Methodol. 2009 Aug 11;9:59. doi: 10.1186/1471-2288-9-59 (PMC3224695; doi:10.1186/1471-2288-9-59)
Supplement: Additional file 1 — Dimensions of difference. Ranging from subjective idealism through objective idealism and critical realism to scientific realism to naïve realism [file 1471-2288-9-59-S1.doc]

FIGURE 1: **Dimensions of difference**

**Ranging from subjective idealism through objective idealism and critical realism to scientific realism to naïve realism [41, p45-46].**

- **Subjective idealism: there is no single shared reality independent of multiple alternative human constructions**
- **Objective idealism: there is a world of collectively shared understandings**
- **Critical realism: knowledge of reality is mediated by our perceptions and beliefs**
- **Scientific realism: it is possible for knowledge to approximate closely an external ‘reality’**
- **Naïve realism maintains that reality exists independently of human constructions and can be known directly.**

**Epistemology**

**Idealist_____________________________________________________________________________________________________________Realist**

| **Meta-narrative** | **CIS** | **Meta-study** | **Meta-ethnography** | **Grounded theory** | **Thematic synthesis** | **Textual narrative synthesis** | **Framework synthesis** | **Ecological triangulation** |
| --- | --- | --- | --- | --- | --- | --- | --- | --- |
| Subjective idealism | Subjective idealism | Subjective idealism | Objective idealism | Objective idealism | Critical realism | Critical realism | Critical realism | Scientific realism |

**The question the method was originally designed to answer (examples are illustrative, not exhaustive):**

| **Meta-narrative** | **CIS** | **Meta-study** | **Meta-ethnography** | **Grounded theory** | **Thematic synthesis** | **Textual narrative synthesis** | **Framework synthesis** | **Ecological triangulation** |
| --- | --- | --- | --- | --- | --- | --- | --- | --- |
| Diffusion of innovation in healthcare systems [32] | Access to healthcare by vulnerable people [34] | The insider experience of chronic illness [28] | Desegregation and interracial education [8]; Lay experiences of diabetes and diabetes care [12]; experiences of resisting medicines [13] | Courage among individuals with long-term health problems [18]; women’s experience of domestic violence [16] | Children’s/young people’s experiences of healthy eating [25] | Barriers to, and facilitators of, health and health behaviour among young people [26] | Involving the public in research [41] | What works for youth with disabilities [38] |

**Approach to quality assessment**

**Non-criteria-based _______________________________________________________________________________________________Criteria-based**

| **Meta-study** | **CIS** | **Grounded theory** | **Meta-ethnography** | **Meta-narrative** | **Textual narrative synthesis** | **Framework synthesis** | **Thematic synthesis** | **Ecological triangulation** |
| --- | --- | --- | --- | --- | --- | --- | --- | --- |
| All relevant studies included; only non-qualitative research excluded | Quality of research judged as the extent to which it informs theory | Quality assessment only discussed in terms of ‘a personal reaction note’ being made on each study about the context, quality and usefulness of each study | Noblit and Hare don’t discuss quality assessment; a later meta-ethnography used an amended version of CASP but only referred to studies being excluded on the basis of lack of relevance or because they weren’t qualitative | Studies evaluated for validity and robustness of methods; sample size and power; validity of conclusions | Seven quality criteria common to sets of criteria proposed by four research groups for qualitative research plus three additional criteria relating to whether studies used appropriate methods for helping people express their views | Ten criteria used: two on quality and reporting of sampling methods, four to the quality of the description of the sample in the study, two to the reliability and validity of the tools used to collect data and one on whether studies used appropriate methods for helping people to express their views | 12 criteria used: five related to reporting aims, context, rationale, methods and findings; four relating to reliability and validity; three relating to the appropriateness of methods for ensuring that findings were rooted in participants’ own perspectives | Adapts the Design and Implementation Assessment Device (DIAD) Version0.3 (a quality assessment tool for quantitative research). Excludes ‘poor quality’ studies |

**Extent of iteration**

**Iteration________________________________________________________________________________________ __________________No iteration**

| **Meta-narrative** | **Meta-study** | **CIS** | **Meta-ethnography** | **Grounded Theory** | **Thematic synthesis** | **Framework synthesis** | **Ecological triangulation** | **Textual narrative synthesis** |
| --- | --- | --- | --- | --- | --- | --- | --- | --- |
| Iteration occurs during every part of the process | Iteration occurs during the data collection stage of meta-data-analysis and during synthesis | Iteration occurs during the searching; not clear whether iteration occurs during the rest of the review process | Iteration occurs during the synthesis stage | Iteration occurs during the synthesis stage | Some iteration at coding and synthesis stages | Iterative literature searching; a priori coding | Not clear | Not clear |

**Problematising the literature**

| **Meta-narrative** | **CIS** | **Meta-study (meta-method, meta-theory)** | **Meta-ethnography** | **Grounded Theory** | **Thematic synthesis** | **Framework synthesis** | **Ecological triangulation** | **Textual narrative synthesis** |
| --- | --- | --- | --- | --- | --- | --- | --- | --- |
| Yes | Yes | Yes | No | No | No | No | No | No |

**Going ‘beyond’ the primary studies**

| **Textual narrative synthesis** | **Ecological triangulation** | **Framework synthesis** | **Meta-ethnography** | **Grounded Theory** | **Thematic synthesis** | **Meta-narrative** | **CIS** | **Meta-study** |
| --- | --- | --- | --- | --- | --- | --- | --- | --- |
| Translation | Translation | Translation | Transformation | Transformation | Transformation | Transformation | Transformation | Transformation |

**Synthetic product**

| **Thematic synthesis** | **Textual narrative synthesis** | **Framework synthesis** | **Ecological triangulation** | **Meta-study** | **Meta-narrative** | **CIS** | **Grounded Theory** | **Meta-ethnography** |
| --- | --- | --- | --- | --- | --- | --- | --- | --- |
| Directly informs policy makers and practitioners | Directly informs policy makers and practitioners | Directly informs policy makers and practitioners | Directly informs policy makers and practitioners | Requires further interpretation by policy makers and practitioners | Requires further interpretation by policy makers and practitioners | Requires further interpretation by policy makers and practitioners | Requires further interpretation by policy makers and practitioners | Requires further interpretation by policy makers and practitioners |

**Whether the data synthesised are homogeneous or heterogeneous**

**Heterogeneous_________________________________________________________________________________________Homogeneous**

| **Meta-narrative** | **Meta-study** | **CIS** | **Framework synthesis** | **Textual narrative synthesis** | **Thematic synthesis** | **Meta-ethnography** | **Grounded theory** | **Ecological triangulation** |
| --- | --- | --- | --- | --- | --- | --- | --- | --- |
| Heterogeneous | Heterogeneous | Heterogeneous | Heterogeneous | Heterogeneous | Heterogeneous | Homogeneous [8]  Heterogeneous [11, 12, 13] | Homogeneous | Not clear |
